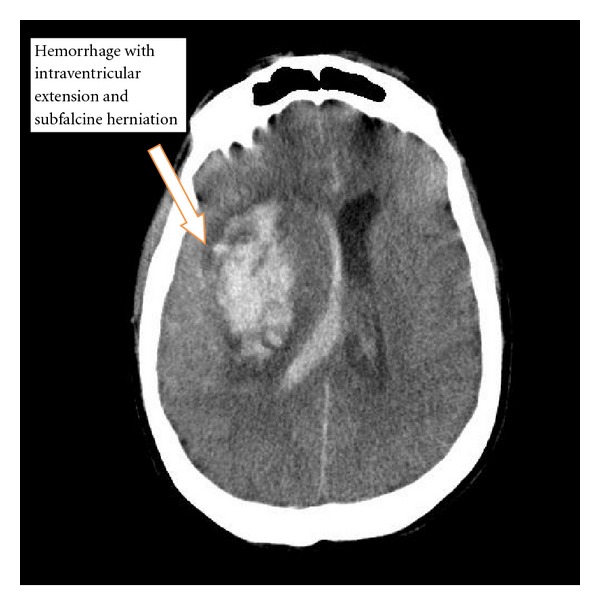# Endocarditis-Induced Mycotic Brain Aneurysm following Right MCA Stroke

**DOI:** 10.1155/2012/606921

**Published:** 2012-05-30

**Authors:** Brandon Allen, Bobby Desai, Michael Falgiani

**Affiliations:** Department of Emergency Medicine, University of Florida College of Medicine, 1329 SW 16th Street, P.O. Box 100186, Gainesville, FL 32610-0186, USA

## Abstract

The diagnosis of cerebrovascular accident is extremely common in emergency medicine; however, CVA resulting from hemorrhage following mycotic brain aneurysm following embolic stroke is extremely uncommon. This case reports such an event.

## 1. Introduction

This is a case report of a patient with right MCA embolic stroke and on hospital day one was found to have endocarditis-induced ruptured mycotic brain aneurysm with herniation. The entity of mycotic brain aneurysm rupture following embolic stroke is exceedingly rare. The sequelae and prognostic ramifications of this condition make it important to examine.

## 2. Case Presentation

A 58-year-old male presents to the Emergency Department via EMS as a possible stroke. Per spouse, he woke up from sleep with left-sided facial droop, slurred speech, and left arm weakness. He had gone to sleep at approximately 10 pm and awoke with the left-sided complaints at approximately 4 am the morning of arrival. He had a past medical history of adrenal adenoma, chronic gastritis, and a vasectomy in 1993. The patient's medications prior to admission were alprazolam, esomeprazole, probiotics, and a daily multivitamin. The patient is aware of his left-sided weakness and complains of heaviness on his left side. He reports no history of alcohol, tobacco, or illicit drug use. The patient's spouse reported that he had been unwell for three months with unexplained weight loss, fever, chills, and loss of appetite. At the time of presentation, various specialists including gastroenterology failed to determine an underlying cause for the patient's complaints besides “anemia of chronic disease.” Furthermore, he was scheduled for an upper and lower endoscopy the day of presentation and was in the process of preparation (cathartics) for that. He has been on a gluten-free diet for elevated tissue transglutaminase and gliadin antibody titers by his endocrinologist. Upon arrival at patient's residence, EMS reported a positive stroke screen with a Cincinnati score of  2, positive for left arm weakness, and left-sided facial droop. Vital signs upon arrival to the Emergency Department were temperature 36.4 degrees Celsius by tympanic reading, pulse 105 beats per minute, blood pressure 116/56 mmHg, respirations 18 breaths per minute, and pulse oximetry 98% on room air. His weight was recorded as 68 kilograms. He was not in any distress but appeared pale and cachectic. His neurologic exam was positive for a left facial droop with weakness of cranial nerves 3, 5, and 7. There was no uvular deviation, and pupils were equal and reactive bilaterally with intact extraocular movements. The upper extremities had symmetric sensation to fine touch with a left pronator drift and 3/5 strength in the proximal and distal muscle groups in comparison to the right. There was no dysmetria. The lower extremities had 5/5 strength bilaterally and absent Babinski reflexes. His NIH stroke scale score was 6. The rest of his physical exam was unremarkable.

**Figure 1:**
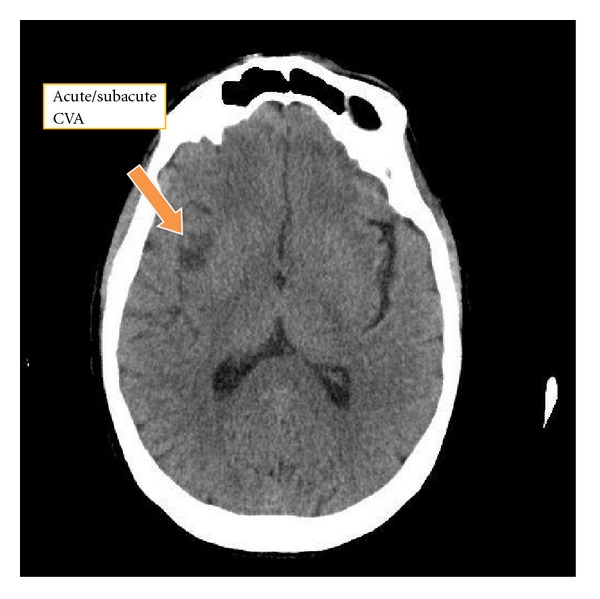


**Figure 2:**
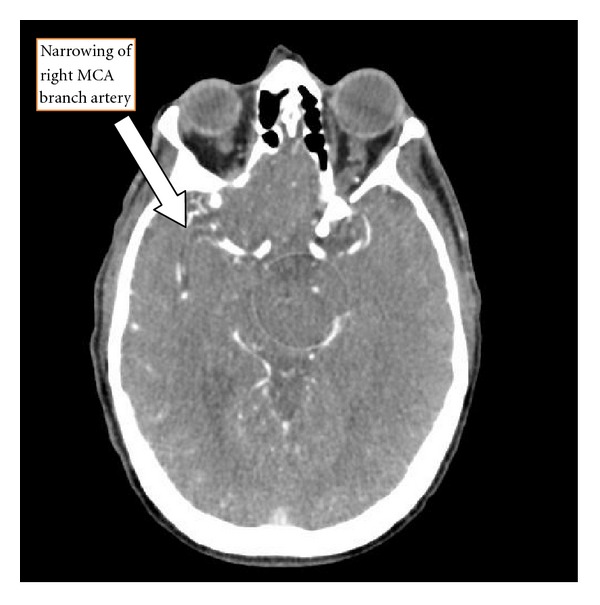


**Figure 3:**
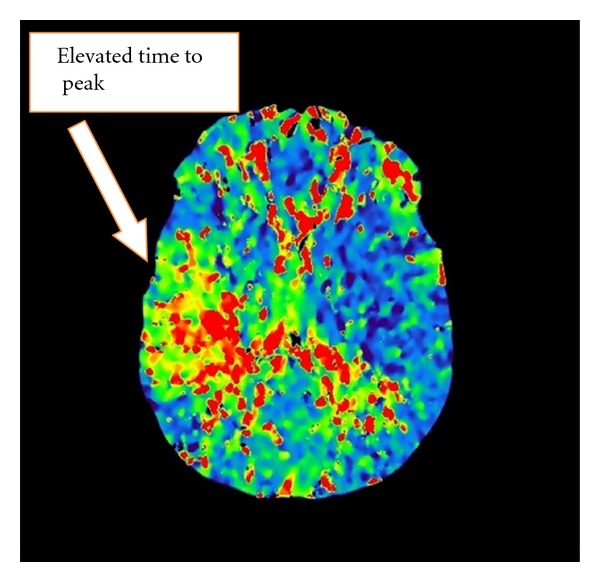


Initial labs included a metabolic panel with measured sodium of 131 mmol/L and blood glucose of 125 mg/dL but were otherwise normal. The complete blood count showed a hemoglobin of 9.6 g/dL and a hematocrit of 29.9%. The white blood cell count was 12.5 thou/cu mm with 78.8% neutrophils and no bands. Cardiac enzymes and urinalysis were both normal. The point-of-care INR was 1.5. Stroke alert protocol imaging included a CT angiogram of the head and neck with and without contrast and postprocedure multiplanar reconstructions. These revealed acute/subacute CVA in the right insula cortex and right basal ganglia (Figure 1) as well as significant narrowing of the proximal M1 branch of the right middle cerebral artery (MCA) secondary to either embolus or atherosclerotic disease (Figure 2). Perfusion images demonstrate elevated time to peak in the posterior right MCA distribution with normal capillary transit time and normal cerebral blood volume consistent with compensated collateral blood flow (Figure 3).

The patient received 300 mg aspirin per rectum and IV fluids prior to admission to the neuro-ICU. The patient was deemed an unsuitable candidate for thrombolysis as onset was estimated approximately 6 hours prior to ED arrival. Neurosurgery was consulted and decided that the risks of endovascular therapy did not outweigh the benefits for the patient.

Followup the next day revealed that the patient had an echocardiogram that showed a mass on the posterior leaflet of the mitral valve with mitral regurgitation. Following echocardiogram, the patient had an acute sudden deterioration in his mental status and displayed respiratory compromise. On examination, his pupils were “blown” (enlarged) bilaterally and he was emergently intubated for airway protection. His emergent noncontrast CT of the head (Figure 4) showed a subcortical hemorrhage with intraventricular extension and subfalcine herniation. Neurosurgery emergently brought the patient to the operating room with a postoperative diagnosis of right intracranial hemorrhage secondary to ruptured mycotic MCA aneurysm. On postoperative day one, the family decided to withdraw care and the patient expired.

## 3. Discussion

Infective endocarditis as an etiology for stroke and mycotic aneurysm is well documented in the literature. Sonneville et al. report that neurologic events are the most frequent complications in infective endocarditis patients requiring intensive care unit admission. Functional independence is lost in up to one-third of these patients, and neurologic failure is a major determinant of mortality [1]. Mycotic aneurysms represent a small but extremely dangerous subset of embolic complications. They occur most frequently in the intracranial arteries and have a particular predilection for the middle cerebral artery and its branches [2]. They result from septic embolization to the arterial vasa vasorum, with subsequent spread of infection and weakening of the vessel wall. Mycotic aneurysms tend to develop at arterial branch points, which are a common site of embolic impaction. The overall mortality rate among IE patients with intracranial mycotic aneurysms is 60 percent and approaches 80 percent if rupture occurs [3]. More recently, Regelsberger et al. report that it is often a fatal clinical course with a mortality rate reaching 90% [4].

The diagnosis of infective endocarditis (IE) must be made as soon as possible to initiate antimicrobial therapy and identify patients at high risk for complications who may be best managed by early surgery. Cerebral complications make the timing of cardiac surgery difficult. Stroke complicates the outcome of left-sided IE in 20–40% of cases and is associated with poor outcome. The risk of stroke in IE falls rapidly after the initiation of effective antimicrobial therapy [5]. In Tornos' study of IE from *S. aureus*, the risk of stroke with native valve endocarditis was 22%; all were ischemic at onset, and 57% became hemorrhagic in the absence of anticoagulation [6]. A large retrospective study by Anderson et al. found fifty-two percent of patients with stroke and IE died within 1 year of admission [7].

## Figures and Tables

**Figure 4:**